# A general method for handling missing binary outcome data in randomized controlled trials

**DOI:** 10.1111/add.12721

**Published:** 2014-11-10

**Authors:** Dan Jackson, Ian R White, Dan Mason, Stephen Sutton

**Affiliations:** 1 Medical Research Council Biostatistics Unit, Cambridge Institute of Public HealthCambridge, UK; 2 University of Cambridge, Behavioural Science GroupCambridge, UK

**Keywords:** Last observation carried forward, missing data, missing not at random, Russell Standard, sensitivity analysis, smoking cessation trials

## Abstract

**Aims:**

The analysis of randomized controlled trials with incomplete binary outcome data is challenging. We develop a general method for exploring the impact of missing data in such trials, with a focus on abstinence outcomes.

**Design:**

We propose a sensitivity analysis where standard analyses, which could include ‘missing = smoking’ and ‘last observation carried forward’, are embedded in a wider class of models.

**Setting:**

We apply our general method to data from two smoking cessation trials.

**Participants:**

A total of 489 and 1758 participants from two smoking cessation trials.

**Measurements:**

The abstinence outcomes were obtained using telephone interviews.

**Findings:**

The estimated intervention effects from both trials depend on the sensitivity parameters used. The findings differ considerably in magnitude and statistical significance under quite extreme assumptions about the missing data, but are reasonably consistent under more moderate assumptions.

**Conclusions:**

A new method for undertaking sensitivity analyses when handling missing data in trials with binary outcomes allows a wide range of assumptions about the missing data to be assessed. In two smoking cessation trials the results were insensitive to all but extreme assumptions.

## Introduction

Missing outcome data are a common problem in randomized controlled trials. In this paper we focus on trials where the end-point of interest is a single binary outcome. Binary outcome measures are widely used in trials for smoking, alcohol and drug misuse where the treatment goal is abstinence 1–4.

In smoking cessation trials, participants who do not report their smoking status at follow-up are often assumed to be smoking 5–8, and the Russell Standard 9,10 requires this. Because smoking cessation trials have this standard approach for handling missing outcome data, we use smoking as our example and incorporate the Russell Standard into our methods. However, our method is applicable to all trial areas where binary outcome data are collected; for example, Maisel *et al*. 2 found that most studies in their meta-analysis of treatments for alcohol-use disorders considered dropouts to have relapsed. Based on an informal review, we estimate that around 80% of reports of smoking cessation trials make the ‘missing = smoking’ assumption.

There are two distinct arguments for treating missing values as smoking. First, we may define a composite outcome of ‘either smoking or missing value’. We are then making no assumption about the missing data, but at the cost of addressing a question with little clinical interest, as health benefit derives from quitting smoking, even if this is not reported. This paper instead considers smoking/quitting as the outcome of main clinical interest. With this outcome, treating missing values as smoking corresponds to making the assumption that any individual with a missing value is smoking. This assumption must be plausible to justify the analysis. Foulds *et al*. 11 provide some relevant evidence: they telephoned study participants who missed appointments in a hospital-based smoking cessation trial and reported that they all had resumed smoking.

A related, but different, approach is last observation carried forward (LOCF). LOCF imputes any missing data with the last observed value, but is widely criticized 12,13. A similar idea is baseline observation carried forward (BOCF), which assumes that participants with missing data have reverted to their baseline behaviour and replaces missing data with baseline values. If the outcome is only measured once after randomization, LOCF is the same as BOCF. In smoking cessation trials where all participants are smoking at baseline BOCF is the same as ‘missing = smoking’, but in our iQuit example (Example 2: the iQuit trial) some participants met the trial criteria to be considered not smoking at baseline.

Missing not at random (MNAR) means that the probability of an outcome being observed or not depends on the unobserved outcomes 14. This is entirely reasonable in the context of smoking cessation trials, because it is plausible that there is an association between participants' smoking status and the probability that this status is observed. In particular, the ‘missing = smoking’ assumption is one example of an MNAR model. A recent paper by Hedeker *et al*. 5, whose methods were subsequently adopted by Smolkowski *et al*. 7, uses logistic regression to model the association between the study outcome and missingness, and uses the outcome at a previous time-point in this regression to predict the missing values; treatment effects are estimated using multiple imputation. Under the assumption that data are missing at random (MAR), we assume instead that the participants' missing outcomes are independent of the missing data indicator (see below), given the covariates and any observed outcomes. It is important to make it clear which covariates are being used in this definition. We perform analyses assuming two versions of MAR: using randomized group alone or also using an additional binary covariate.

In practice, it is very difficult to say which of the above assumptions about missing data is correct. Our approach allows the analyst to investigate a range of plausible possibilities, as we illustrate in the Supporting information appendix that shows our results for the iQuit data.

In this paper we describe a model for missing outcome abstinence data that extends the procedure suggested by Hedeker *et al*. 5 in the following ways. First, unlike Hedeker *et al*., we include treatment group in the ‘imputation’ model (our model 1 below includes the treatment group as a covariate). This is important, because it allows the possibility of different missing data mechanisms to operate in different treatment arms, which may be plausible when participants are subject to very different treatments and so fail to provide data for different reasons. Secondly, Hedeker *et al*. used formulae for standard errors which assume that the unidentifiable parameters are estimated using the data. We show how to obtain standard errors which take into account the fact that some parameters are provided by the analyst, rather than estimated from the data. Thirdly, we provide a spreadsheet which enables calculation of intervention effects and standard errors to be performed quickly without resorting to multiple imputation.

## Statistical model

We present our model in terms of smoking cessation trials, so that the binary end-point of interest is smoking cessation. Hence, assuming ‘missing = smoking’ follows the Russell Standard 9,10. The corresponding assumption in trials of treatments for alcohol or substance misuse would be ‘missing = drinking’ or ‘missing = using’. We embed the Russell standard in our method, so the analyst is free to move away from this as much, or as little, as desired.

Smoking status at a previous time-point is commonly measured and may be useful in predicting participants' smoking status at the end of the trial. We denote this variable by *X*, so that *X* = 0 means that the participant abstained from smoking at this time-point and *X* = 1 otherwise, but more generally *X* may denote any binary covariate. If *X* measures a quantity other than the outcome at a previous time-point then the interpretation of some models as corresponding to LOCF below is lost. We assume that data on *X* are complete. If no such variable *X* is available, we drop this variable from our model.

We assume a single binary outcome *Y*, which takes the value 1 if the participant is smoking and 0 if they have abstained from smoking at the end of the trial. We use *R* to denote the missing data indicator, so that *R* = 1 means that *Y* is observed and *R* = 0 means that it is missing. We let *Z* denote the randomized group and assume there are two such groups; *Z* = 0 indicates that the participant was randomized to the control group and *Z* = 1 indicates the treatment group.

We follow, but extend, Hedeker *et al*. 5 by allowing a treatment group effect in their model (6) and assume that:
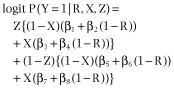
1

The parameters β_1_, β_3_, β_5_ and β_7_ denote the log-odds of participants smoking in particular subsets where smoking status is observed, as summarized in Table 1. Hence β_1_, β_3_, β_5_ and β_7_ are identifiable, and are estimated as the log-odds of smoking in each *(X*, *Z)* group, using data from participants whose smoking status is known.

**Table 1:** Summary of odd-numbered β parameters: each parameter denotes the log-odds of smoking in a particular subset where smoking status is observed

Parameter	Previous (X)	Treatment group (Z)	Observed(R)
β_1_	0 (not smoking)	1 (treatment)	1 (yes)
β_3_	1 (smoking)	1 (treatment)	1 (yes)
β_5_	0 (not smoking)	0 (control)	1 (yes)
β_7_	1 (smoking)	0 (control)	1 (yes)

The parameters β_2_, β_4_, β_6_ and β_8_ denote the log-odds ratio of smoking for particular subsets of participants, comparing those with missing outcomes to those with observed outcomes, as summarized in Table 2. The parameters β_2_, β_4_, β_6_ and β_8_ relate the success of participants with missing outcomes to their counterparts whose outcomes are observed. Hence, the data provide no information about these parameters and so they will be held fixed to particular values in the context of a sensitivity analysis. A range of values will be chosen to correspond to the kinds of assumptions described in the Introduction and other values that are considered plausible. This kind of approach has been suggested previously in the context of meta-analysis by Higgins *et al*. 15, who describe the exponential of parameters similar to those in Table 2 as informatively missing odds ratios (IMORs).

**Table 2:** Summary of even-numbered β parameters: each parameter denotes the log–odds ratio of smoking, comparing participants with missing relative to those with observed outcomes, in a particular subset of participants

Parameter	Previous (X)	Treatment group (Z)
β_2_	0 (not smoking)	1 (treatment)
β_4_	1 (smoking)	1 (treatment)
β_6_	0 (not smoking)	0 (control)
β_8_	1 (smoking)	0 (control)

### Interpretation of the even-numbered β parameters

If all of β_2_, β_4_, β_6_ and β_8_ are zero, then in each (*X*, *Z*) group the distribution of the outcome *Y* is the same for both *R* = 0 and *R* = 1. Hence, *Y* and *R* are conditionally independent and β_2_ = β_4_ = β_6_ = β_8_ = 0 corresponds to MAR. Hence, the MAR assumption is a special case of our model 1. If we make the weaker assumption that β_2_ = 0, for example, then we assume that data in the (*X* = 0, *Z* = 1) group are MAR. Similarly, if we assume β_2_ = β_4_ = 0 then we assume the treatment group data are MAR, and so on.

Another possible assumption is LOCF. If we let β_2_ be large and negative, then missing values in the (*X* = 0, *Z* = 1) group are taken to be non-smokers, and so *Y* = 0 = *X*, and we have LOCF in this group. If instead we let β_2_ be large and positive then we assume ‘missing = smoking’ in this group. Hence β_2_ = 0 can be seen to be a compromise between LOCF and the ‘missing = smoking’ assumption for the (*X* = 0, *Z* = 1) group. Conversely, large and positive values of β_4_ correspond to both LOCF and ‘missing = smoking’ in the (*X* = 1, *Z* = 1) group. Similar arguments apply for β_6_ and β_8_. A range of possibilities is summarized in Table 3. The infinite values for parameters in Table 3 correspond to the type of assumptions often made when encountering missing data. We allow the assumptions made about the missing outcome data to differ in the two treatment groups, although making the same assumption in both groups, so that β_2_ = β_6_and β_4_ = β_8_ may often be considered plausible.

**Table 3:** Some possible assumptions about the missing outcome data, expressed in terms of the even-numbered β parameters. Assumptions for a particular group (*X*, *Z*) are shown only for the group (*X* = *0*, *Z* = *1*) for brevity

Assumption				Interpretation
β_2_	β_4_	β_6_	β_8_	
0	0	0	0	All data are MAR
0	–	–	–	Data in the (*X* = *0*, *Z* = *1*) group are MAR
0	0	–	–	Data in the treatment group are MAR
–	–	0	0	Data in the control group are MAR
−∞	+∞	−∞	+∞	All data LOCF
−∞	–	–	–	Data in the (*X* = *0*, *Z* = *1*) group LOCF
−∞	+∞	–	–	Data in the treatment group LOCF
–	–	−∞	+∞	Data in the control group LOCF
+∞	+∞	+∞	+∞	All data missing = smoking
+∞	–	–	–	Data in the (*X* = *0*, *Z* = *1*) group missing = smoking
+∞	+∞	–	–	Data in the treatment group missing = smoking
–	–	+∞	+∞	Data in the control group missing = smoking

MAR = missing at random; LOCF = last observation carried forward.

+∞ = Large and positive; −∞ large and negative.

## Estimation

### Estimating the intervention effect

The target parameter for inference is the intervention effect:
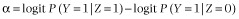
2which is the log odds ratio measuring the association between treatment group and smoking. *X* plays an important role as a predictor for any missing outcome data but does not contribute to the definition of the target parameter. Using the definition of *Y* adopted here, a negative *α* indicates a beneficial treatment and so the corresponding odds ratio of <1 also indicates a treatment benefit.

The estimation procedure is conceptually simple. First we select the four values of the even numbered β sensitivity parameters that we wish to use. We then estimate the parameters β_1_, β_3_, β_5_and β_7_. As an example, we focus on the 131 participants in the iQuit trial (see Example 2: the iQuit trial) who are in the treatment group (*Z* = 1) and are considered not smoking at baseline (*X* = 0). Of the 65 participants in this group who provide outcome data (*R* = 1), 41 (63%) were smoking at the end of the trial and so we estimate β_1_ to be logit(41/65) = 0.536.

Having estimated all four odd-numbered β parameters, we then use equation 1 to estimate the probability of smoking for each combination of *R*, *X* and *Z*. In our example, suppose we choose β_2_ = 1. Then we estimate the log odds of smoking among non-responders in the treatment group considered not smoking at baseline (*Z* = 1, *X* = 0, *R* = 0) as 0.536 + 1 = 1.536, so the proportion smoking in this group is 82%. Similarly, for the 746 participants who are in the treatment group and are considered smoking at baseline (*Z* = 1, *X* = 1), 286 provided outcome data of whom 230 (80%) were smoking at follow-up; and supposing β_4_ = 1 we estimate that 92% of the 460 who did not provide outcome data were smoking at follow-up.

Having estimated all the above probabilities, we use the law of total probability to estimate the probability of smoking in each treatment group. In our example, the overall probability of smoking (combining responders and non-responders and combining those not smoking and smoking at baseline) in the treatment group is (65 × 63% + 66 × 82% + 286 × 80% + 460 × 92%)/(65 + 66 + 286 + 460) = 85%. The procedure is repeated in the control arm leading to an estimate α of the intervention effect. A mathematical formula that is equivalent to following this procedure is given in the Supporting information.

### Standard errors

We have developed an approximate standard error of the estimated treatment effect. This is based on the delta method. As both the delta method and the standard formula for the variance of an empirical log odds ratio are based on first-order Taylor Series expansions, these approximate standard errors agree exactly when infinite even β are used and when data are imputed following the same assumption (Table 3). Therefore both the estimate α (see section Estimating the intervention effect) and its standard error agree with the results obtained from simple imputation when the model is equivalent to assuming ‘missing = smoking’ or ‘LOCF’ in the trial arms. A document containing the proof of this for the LOCF case, with a full derivation of the standard error, is available in the Supporting information. Both the estimates and the standard errors take the β_2_, β_4_, β_6_ and β_8_ parameters as fixed and no uncertainty in their value is accounted for when performing inference using a particular set of these sensitivity parameters. However, the uncertainty in the odd-numbered β parameters, and therefore in the inferred quit rates, is taken into account. A range of plausible values for the sensitivity values should be explored, so that the implications of using different values can be assessed.

### An equivalence between assuming MAR and a complete case analysis

As explained above, if no *X* variable is available we drop this from our model. MAR (where *Z* is now the only covariate) now results in the same inference as a standard complete case logistic regression of *Y* on *Z*.

### Software

Our model and mode of analysis is sufficiently straightforward that all the results can be produced by our purpose-built spreadsheet. Our spreadsheet is provided as part of the Supporting information that accompanies this paper. This spreadsheet can be used to reproduce the results that follow and by those wishing to conduct similar sensitivity analyses for their data. Our spreadsheet also converts the results using the log odds ratio as the measure of treatment effect to results using the risk difference and the log relative risk. This requires changing the logit function in equation 2 to the identify function and the log function for the risk difference and log relative risk, respectively.

## Application to the example data sets

### Example 1: Hedeker's data

This study 4,16 evaluated the effectiveness of adding group-based treatment adjuncts to a smoking cessation intervention. Two types of group adjuncts were compared. We follow Hedeker *et al*. and combine the controls and ‘no-shows’ to provide the ‘control’ group and the two active treatments to provide the ‘treatment’ group, but we also recognize that this is dubious and only provides an illustrative analysis. The pattern of missingness by treatment group is shown in Table 4. There is an appreciably larger proportion of missing outcome data in the control group.

**Table 4:** The pattern of missingness for Hedeker's data

	Control	Treatment	Total
Smoking known	216 (72%)	156 (82%)	372 (76%)
Smoking unknown	83 (28%)	34 (18%)	117 (24%)
Total	299	190	489

We will perform a range of analyses that show how the inferences change depending on the assumptions made about the missing outcome data. We refer to this as a sensitivity analysis.

We first perform the analysis described above (‘An equivalence between assuming MAR and a complete case analysis’), a logistic regression of *Y* on *Z*. This is equivalent to assuming data are MAR, and making no use of the covariate *X*.

In order to illustrate our method, we then make a range of alternative assumptions about the missing outcome data in both treatment groups. Some results are shown in Table 5. ‘Missing = smoking’ imputes only smokers; as the majority of participants whose outcome is known at the end of the trial are smoking, this assumption has little impact in reducing the standard error. LOCF artificially reduces the standard error of the treatment effect because it imputes some non-smokers.

**Table 5:** Results from the sensitivity analysis for Hedeker's data. The notation treatment/control in the Assumption column refers to the assumption made in the treatment and control groups, respectively

β_2_	β_4_	β_6_	β_8_	Assumption	α (Standard error)	OR (95% CI)
–	–	–	–	MAR (ignoring *X*; section 3.3)	−0.35 (0.26)	0.71 (0.43,1.17)
0	0	0	0	MAR (using *X* and *Z*)	−0.33 (0.25)	0.72 (0.44, 1.18)
−∞	+∞	−∞	+∞	LOCF	−0.39 (0.22)	0.68 (0.44, 1.03)
+∞	+∞	+∞	+∞	‘Missing = smoking’	−0.48 (0.25)	0.62 (0.38, 1.01)
0	0	−∞	+∞	MAR/LOCF	−0.21 (0.23)	0.81 (0.51, 1.28)
0	0	+∞	+∞	MAR/'missing = smoking’	−0.74 (0.25)	0.48 (0.29, 0.78)
−∞	+∞	0	0	LOCF/MAR	−0.51 (0.24)	0.60 (0.38, 0.95)
−∞	+∞	+∞	+∞	LOCF/'missing = smoking’	−0.92 (0.23)	0.40 (0.25, 0.63)
+∞	+∞	0	0	‘Missing = smoking'/MAR	−0.08 (0.25)	0.93 (0.57, 1.52)
+∞	+∞	−∞	+∞	‘Missing = smoking'/LOCF	0.05 (0.23)	1.05 (0.67, 1.65)

MAR = missing at random; LOCF = last observation carried forward; OR = odds ratio; CI = confidence interval.

In order to further illustrate our method, additional results can be obtained by making alternative assumptions in each (*X*, *Z*) group, as shown in Table 3, or by assuming finite, but non-zero, values of even β. For example, Hedeker *et al*. 4 consider stratified odds ratios of 1, 2 and 5, which correspond to 0 (MAR), log(2) and log(5) for the even β parameters. A similar approach can be adopted here by producing results similar to those in Table 5 but where infinite values of β are replaced by, for instance, log(2). Infinite values of β that correspond to ‘missing = smoking’ or LOCF in Table 5 are now replaced with more moderate assumptions: that participants with missing data are more likely to be smoking, or continue to provide their last observed value, than their counterparts with complete outcome data, respectively. The results are shown in Table 6 and follow similar trends as in Table 5, but all estimates bear much more resemblance to those from a MAR analysis, as expected. Overall, we conclude that the inferences from these data are not very sensitive to the assumptions made about the missing data.

**Table 6:** Further results from the sensitivity analysis of Hedeker's data. Standard errors of *â* and the confidence interval (CI) for the odds ratio (OR) are in parentheses

β_2_	β_4_	β_6_	β_8_	α *(Standard error)*	*OR (95% CI)*
−log(2)	log(2)	−log(2)	log(2)	−0.37 (0.25)	0.69 (0.43, 1.12)
log(2)	log(2)	log(2)	log(2)	−0.39 (0.25)	0.68 (0.41, 1.11)
0	0	−log(2)	log(2)	−0.33 (0.25)	0.72 (0.44, 1.17)
0	0	log(2)	log(2)	−0.49 (0.25)	0.61 (0.37, 1.01)
−log(2)	log(2)	0	0	−0.37 (0.25)	0.69 (0.42, 1.13)
−log(2)	log(2)	log(2)	log(2)	−0.53 (0.25)	0.59 (0.36, 0.97)
log(2)	log(2)	0	0	−0.23 (0.25)	0.79 (0.48, 1.30)
log(2)	log(2)	−log(2)	log(2)	−0.23 (0.25)	0.79 (0.49, 1.29)

### Example 2: the iQuit trial

The iQuit trial 17 is an internet-based randomized controlled trial conducted among the general population of smokers seeking help from web-based resources. It assessed the effect on smoking cessation rates of self-help materials tailored to individual smoker characteristics compared with generic self-help materials. We used the same methodology as in the previous example for analysing these data. However, here we elicit plausible values for the sensitivity parameters and use only these values. See the Supporting information for further details.

## Implications for the design of trials

Perhaps the most important and obvious implication of the results obtained for our two examples is that we should attempt to minimize the amount of missing data 18. If some missing outcome data are inevitable then we suggest four possible strategies to reduce their impact.

First, collecting data on the reasons for missing data is important in order to determine the kinds of assumptions that are plausible. For example, if data are missing because a participant has moved away then the assumption that their data are MAR may be entirely plausible.

Secondly, collecting outcome data from participants at intermediate time-points is extremely valuable. Any additional outcome measurements provide more observed data to condition on in the definition of MAR and so make this more plausible 19–21. A likelihood-based MAR analysis using all outcome data could then be performed using a mixed-effects model, where the dependence between outcomes from the same individual are taken into account. This type of analysis also allows participants who provide an outcome at some point, but not necessarily the final outcome, to contribute to the analysis. However, this type of approach is more difficult with binary, rather than continuous, outcome data 22.

Thirdly, recording the number of attempts made to obtain final outcome data, or possibly some other measure of the difficulty in obtaining data, is useful because this provides a means to determine how far the data depart from MAR 23–25. Hence, some of the possibilities examined in the sensitivity analyses performed here might be ruled out because they are not supported by the number of attempts data and modelling.

Finally, covariates related to missingness can be collected and used in the analysis model to make the MAR assumption more plausible.

## Discussion

Analysis of a randomized trial in addictions should start with a ‘main analysis’ which is valid under a pre-specified assumption about the missing data 26. Often the assumption underlying the main analysis is ‘missing = smoking’. This assumption is thought to be conservative, but this need not be the case; further, it is questionable if conservatism is desirable, because this reduces the probability of successfully detecting an effective treatment. Nelson *et al*. 27 state that Lichtenstein & Glasgow 28 only suggested ‘missing = smoking’ as a conservative approach for estimating abstinence rates (and not for comparing abstinence rates, as we do here).

Because assumptions such as ‘missing = smoking’ are unlikely to be exactly true, sensitivity analysis is essential 26. Our method provides an easy way to perform such sensitivity analysis. First, plausible ranges of values of the even β parameters should be defined, ideally before the data are analysed, as in the Supporting information appendix showing our results for the iQuit data. This Supporting information appendix provides guidance, but not strict guidelines, for those using our method. Then the intervention effect should be computed over these ranges of values of the even β. If the conclusions about the intervention effect are unchanged in all sensitivity analyses then the conclusions may be regarded as robust. The results of the sensitivity analysis may be regarded as an improvement on the results of the main analysis if the ranges of values of the even β are reasonable, because they are more likely to include the true value of the intervention effect. Unfortunately, because the observed data do not give us any information about the missing data, there is usually no way of knowing if any given analysis with missing data is correct. Thus any analysis is only as good as the plausibility of its assumptions: our claim is that sensitivity analysis can achieve greater plausibility by encompassing a wider range of assumptions.

The proposed method is relatively easily implemented. Models with a more complicated data structure *X* to predict missingness are a relatively straightforward extension of our method, where we specify model 1 for all combinations of *X*, *R* and *Z*, and we sum over all combinations of *X* and *R* for each treatment group separately in equation 2. If *X* were continuous we would instead integrate over *X*. However, with richer and more complicated data comprising *X*, one would almost certainly have to assume common β parameters in model 1 for some of the resulting groups of participants, in order to reduce the amount of modelling required. Further treatment groups can be added easily and pairwise treatment comparisons can be made using the estimate for each treatment pair.

We have assumed that the target for inference is the unadjusted log odds ratio of the association between the outcome and the randomized group. We anticipate that this will commonly be the case for randomized controlled trials with binary outcomes. However, extending the method to allow alternative measures of treatment effect, such as risk differences or relative risks, is straightforward and our spreadsheet also estimates these two measures. We proposed our method for estimating α because it reduces to the usual unadjusted estimates that are obtained when imputing values assuming ‘missing = smoking’ and LOCF, when appropriate sensitivity parameters are used.

Alternative estimators of treatment effect are also possible; for example, one may wish to adjust the treatment effect for baseline covariates. When the even-numbered β parameters are infinite the proposed method is equivalent to imputing successes or failures. Hence, adjusted analyses could be performed on a variety of imputed data sets in the context of a sensitivity analysis. However, we leave the best way to perform an analysis with finite sensitivity parameters and adjusted treatment effects as an open question. If the outcome is recorded at more than one follow-up time-point, then analyses assuming that particular groups of participants' data are MAR, or instead using ‘missing = smoking’ or LOCF for some or all groups, may also be performed by imputing outcomes where appropriate and then fitting a (possibly generalized) mixed-effects model. Hence, a sensitivity analysis akin to the one in Table 5 is computationally straightforward. The best way to generalize our full model for a longitudinal trial remains an open research question, but pattern mixture modelling 12 provides a wide range of possibilities.

To summarize, by using our method triallists working in all areas can perform a thorough sensitivity analysis and assess the consequences of a wide range of assumptions for the estimated treatment effect. These assumptions include the Russell Standard for smoking cessation trials and we hope that this paper will encourage triallists to consider using our approach.

### Declaration of interests

The iQuit trial was supported by funding from Cancer Research UK reference no. C4496/A7775. D.J. and I.R.W. are employed by the UK Medical Research Council [Unit Programme number U105260558].

## Supporting Information

Appendix S1 Spreadsheet containing iQUIT data.

Appendix S2 Estimating the intervention effect: mathematical details.

Appendix S3 Further details of the spreadsheet.

Appendix S4 Example 2: the iQuit trial.
