# Supplementary material for: A general method for handling missing binary outcome data in randomized controlled trials
Source: Addiction. 2014 Nov 10;109(12):1986–93. doi: 10.1111/add.12721 (PMC4241048; doi:10.1111/add.12721)
Supplement: Appendix S1 — Spreadsheet containing iQUIT data. [file add0109-1986-SD1.pdf]

Supplementary Materials for "An improved method for handling missing binary outcome data in randomised controlled trials"

Dan Jackson, Ian R White, Dan Mason and Stephen Sutton

March 7, 2014

1 The target parameter

The target parameter for inference is the unadjusted log odds ratio as given in equation (2) in the supplementary materials.

$$\alpha = \text{logit}\left(\sum_{i=1}^{2}\sum_{j=1}^{2} P(X=i, R=j|Z=1)P(Y=1|X=i, R=j, Z=1)\right) - \text{logit}\left(\sum_{i=1}^{2}\sum_{j=1}^{2} P(X=i, R=j|Z=0)P(Y=1|X=i, R=j, Z=0)\right) \quad (1)$$

We obtain an estimate of α as also explained in the paper. We estimate β_1, β_3, β_5 and β_7 using data from participants who provide outcome data. We assume particular fixed values of β_2, β_4, β_6 and β_8.

We can then estimate the $P(Y = 1|X = i, R = j, Z = z)$ terms in (1) using the estimates of the odd numbered β parameters and fixed values of even numbered β parameters. We use the empirical proportions of participants to estimate the $P(X = i, R = j|Z = z)$ terms. We then substitute these estimates into (1) to obtain $\hat{\alpha}$. We require a standard error of $\hat{\alpha}$.

2 Calculating the standard error

We will obtain the standard error of the estimated treatment effect by initially evaluating the variance of the first (treatment effect) term in the estimate of α. That is, we will evaluate

$$\text{Var}\left[\left(\sum_{i=1}^{2}\sum_{j=1}^{2}\hat{P}(X=i,R=j|Z=1)\hat{P}(Y=1|X=i,R=j,Z=1)\right)\right] \quad (2)$$

Since the treatment and control groups are assumed to be independent, the variance of $\hat{\alpha}$ is the sum of this variance and the corresponding control group variance, from which the standard error of $\hat{\alpha}$ can be obtained by taking the square root.

We will use some additional notation. We define $N_{x,r,z}$ as the number of subjects providing $X = x$, $R = r$ and $Z = z$. We define N_z as the number of subjects in the treatment group z. The N_z (and hence the total sample size) are regarded as fixed but the $N_{x,r,z}$ are regarded as random variables. More specifically, the random variables $N_{x,r,1}$ will be modelled using a multinomial distribution. Furthermore all $N_{x,r,z}$ are independent of all estimates of β parameters.

Equation (2) can then be written in terms of six random variables

$$\text{Var}\left[f(N_{0,0,1},N_{0,1,1},N_{1,0,1},N_{1,1,1},\hat{\beta}_1,\hat{\beta}_3)\right] \quad (3)$$

where $f(N_{0,0,1},N_{0,1,1},N_{1,0,1},N_{1,1,1},\hat{\beta}_1,\hat{\beta}_3) =$

$$\text{logit}\left(\frac{N_{0,0,1}\text{expit}(\hat{\beta}_1+\beta_2)+N_{0,1,1}\text{expit}(\hat{\beta}_1)+N_{1,0,1}\text{expit}(\hat{\beta}_3+\beta_4)+N_{1,1,1}\text{expit}(\hat{\beta}_3)}{N_1}\right)$$

2.1 The covariance matrix of these six random variables

Using the observed proportions to estimate the covariance matrix of the multinomial $N_{x,r,z}$ variables we have that the covariance matrix of $(N_{0,0,1}, N_{0,1,1}, N_{1,0,1}, N_{1,1,1})$, $\mathbf{C}_N$, is given by

$$\frac{1}{N_1}\begin{bmatrix} N_{0,0,1}(N_1 - N_{0,0,1}) & -N_{0,0,1}N_{0,1,1} & -N_{0,0,1}N_{1,0,1} & -N_{0,0,1}N_{1,1,1} \\ -N_{0,0,1}N_{0,1,1} & N_{0,1,1}(N_1 - N_{0,1,1}) & -N_{0,1,1}N_{1,0,1} & -N_{0,1,1}N_{1,1,1} \\ -N_{0,0,1}N_{1,0,1} & -N_{0,1,1}N_{1,0,1} & N_{1,0,1}(N_1 - N_{1,0,1}) & -N_{1,0,1}N_{1,1,1} \\ -N_{0,0,1}N_{1,1,1} & -N_{0,1,1}N_{1,1,1} & -N_{1,0,1}N_{1,1,1} & N_{1,1,1}(N_1 - N_{1,1,1}) \end{bmatrix}$$

and the covariance matrix of $(\hat{\beta}_1, \hat{\beta}_3)$, $\mathbf{C}_\beta$, is given by the usual formula for the variance of the logit of a proportion

$$\begin{bmatrix} (1+\exp(\hat{\beta}_1))/(N_{0,1,1}\text{expit}(\hat{\beta}_1)) & 0 \\ 0 & (1+\exp(\hat{\beta}_3))/(N_{1,1,1}\text{expit}(\hat{\beta}_3)) \end{bmatrix}.$$

Hence the covariance matrix of $(N_{0,0,1}, N_{0,1,1}, N_{1,0,1}, N_{1,1,1}, \hat{\beta}_1, \hat{\beta}_3)$ is approximately $\mathbf{C} = \text{diag}(\mathbf{C}_N, \mathbf{C}_\beta)$.

2.2 The delta method

Now that we have the covariance matrix of all six parameters of interest, we can evaluate an approximate variance (3) using the delta method as

$$\text{Var}\left[f(N_{0,0,1}, N_{0,1,1}, N_{1,0,1}, N_{1,1,1}, \hat{\beta}_1, \hat{\beta}_3)\right] \approx t(\bigtriangledown f)\mathbf{C} \bigtriangledown f \qquad (4)$$

where $t(\cdot)$ denotes matrix transpose and $\bigtriangledown f$ is the vector of partial derivatives of f with respect to the six random variables, where all quantities are evaluated at their observed values. We can evaluate $\bigtriangledown f$ as

$$\bigtriangledown f = \frac{1}{N_1\hat{P}(Y=1|Z=1)(1-\hat{P}(Y=1|Z=1))}\times$$

$$\begin{bmatrix} \text{expit}(\hat{\beta}_1 + \beta_2) \\ \text{expit}(\hat{\beta}_1) \\ \text{expit}(\hat{\beta}_3 + \beta_4) \\ \text{expit}(\hat{\beta}_3) \\ N_{0,1,1}\text{expit}(\hat{\beta}_1)/(1+\exp(\hat{\beta}_1)) + N_{0,0,1}\text{expit}(\hat{\beta}_1 + \beta_2)/(1+\exp(\hat{\beta}_1 + \beta_2)) \\ N_{1,1,1}\text{expit}(\hat{\beta}_3)/(1+\exp(\hat{\beta}_3)) + N_{1,0,1}\text{expit}(\hat{\beta}_3 + \beta_4)/(1+\exp(\hat{\beta}_3 + \beta_4)) \end{bmatrix}$$

Hence the variance (3) can be approximated using this vector and (4). The variance of the second (control group) term in the estimate of α is obtained by replacing all quantities with their corresponding control group values. Hence the standard error of $\hat{\alpha}$ can be obtained.

3 Evaluating the standard error in practice

The delta approximation (4) can be evaluated without resorting to matrix multiplication by noting that both $\mathbf{C}$ and $t(\triangledown f) \triangledown f$ are symmetric matrices. Then

$$t(\triangledown f)\mathbf{C} \triangledown f = \text{tr}(t(\triangledown f)\mathbf{C} \triangledown f) = \text{tr}(\mathbf{C}\{\triangledown f t(\triangledown f)\}) = \sum(\mathbf{C} \bigotimes \{\triangledown f t(\triangledown f)\}) \quad (5)$$

where tr denotes the trace operator, $\otimes$ denotes *element by element* matrix multiplication and $\sum$ denotes the summation of all elements in the matrix. Since the entries of $\triangledown f t(\triangledown f)$ and $\mathbf{C}$ are easily evaluated, we can conveniently evaluate the variance (4) from (5). The purpose built spreadsheet evaluates variances, and hence the standard error of $\hat{\alpha}$, in this way.

4 The standard errors when all even numbered β parameters are infinite

When all even numbered β parameters are infinite then we assume that all missing data are either zero or one, depending on whether the corresponding infinite even numbered β parameter is positive or negative. Hence the model is equivalent to imputing missing data in a deterministic way.

From the form of (1) it is clear that the same estimate of treatment effect is obtained from our procedure when using infinite even numbered β parameters as when imputing missing data and calculating the resulting empirical log odds ratio between the outcome and the treatment group. Furthermore the proposed procedure results in the same standard error. Here we will show that this is the case for Last Observation Carried Forward (LOCF) in the treatment group.

To obtain LOCF in the treatment group, we take the limits $\beta_2 \to -\infty$ and $\beta_4 \to \infty$. This results in considerable simplification

$$\bigtriangledown f = \frac{1}{N_1 \hat{P}(Y=1|Z=1)(1-\hat{P}(Y=1|Z=1))} \begin{bmatrix} 0 \\ \text{expit}(\hat{\beta}_1) \\ 1 \\ \text{expit}(\hat{\beta}_3) \\ N_{0,1,1}\text{expit}(\hat{\beta}_1)/(1+\exp(\hat{\beta}_1)) \\ N_{1,1,1}\text{expit}(\hat{\beta}_3)/(1+\exp(\hat{\beta}_3)) \end{bmatrix}$$

We define $Y_{x,1,z}$ as the number of subjects with $X = x$, $R = 1$ and $Z = z$ who provide $Y = 1$. We can then equate $\text{expit}(\hat{\beta}_1)=Y_{0,1,1}/N_{0,1,1}$, $(1+\exp(\hat{\beta}_1))^{-1} = (N_{0,1,1} - Y_{0,1,1})/N_{0,1,1}$, $\text{expit}(\hat{\beta}_3)=Y_{1,1,1}/N_{1,1,1}$ and $(1+\exp(\hat{\beta}_3))^{-1} = (N_{1,1,1} - Y_{1,1,1})/N_{1,1,1}$. Using these identities and evaluating (5) gives, after some te-

dious algebra,

$$\frac{N_1((Y_{0,1,1}+Y_{1,1,1}+N_{1,0,1}/N_1)((N_1-Y_{0,1,1}-Y_{1,1,1}-N_{1,0,1})/N_1)}{(N_1\hat{P}(Y=1|Z=1)(1-\hat{P}(Y=1|Z=1)))^2}$$

The numerator is the product of N_1 and two proportions: the first is proportion of subjects who provide $Y = 1$ in the treatment group if all subjects with $X = 1$ and $R = 0$ in this group are imputed as if giving $Y = 1$ and if all subjects with $X = 0$ and $R = 0$ are imputed as $Y = 0$, ie we impute as in LOCF. The second proportion is the corresponding proportion of subjects who do not give $Y = 1$, ie give $Y = 0$, if we impute as in LOCF. The probabilities in the denominator are estimated using these same proportions and hence some cancelation occurs and we obtain

$$\frac{1}{(N_1\hat{P}(Y=1|Z=1)(1-\hat{P}(Y=1|Z=1)))}$$

This formula is simply the usual variance of the logit of a proportion and hence is the same contribution to the standard error of $\hat{\alpha}$ is as obtained as when imputing the treatment group data as LOCF and treating it as real data.

Similar arguments can be made for the control groups contribution to the variance of $\hat{\alpha}$, and simpler but similar calculations show that an analogous result is obtained for "missing=smoking", where we instead take the limits $\beta_2 \to \infty$ and $\beta_4 \to \infty$.
