# Supplementary material for: A general method for handling missing binary outcome data in randomized controlled trials
Source: Addiction. 2014 Nov 10;109(12):1986–93. doi: 10.1111/add.12721 (PMC4241048; doi:10.1111/add.12721)
Supplement: Appendix S2 — Estimating the intervention effect: mathematical details. [file add0109-1986-SD2.docx]

**Estimating the intervention effect: mathematical details**

The target parameter for inference is the intervention effect

$$\alpha=\text{ logit P}\left(\text{Y}=\text{1 }|\text{ Z}=1\right)\text{ }-\text{ logit P}\left(\text{Y}=\text{1 }|\text{ Z}=0\right)$$

which is the log odds ratio measuring the association between treatment group and smoking. *X* plays an important role as a predictor for any missing outcome data but does not contribute to the definition of the target parameter. Using the definition of *Y* adopted here, a negative $\alpha$ indicates a beneficial treatment. We can obtain an expression for $\alpha$ using the law of total probability

$\begin{matrix} \alpha=\text{logit}\left(\sum_{i=1}^{2} \sum_{j=1}^{2} P(X=i,R=j|Z=1)P(Y=1|X=i,R=j,Z=1)\right)- \\ \text{logit}\left(\sum_{i=1}^{2} \sum_{j=1}^{2} P(X=i,R=j|Z=0)P(Y=1|X=i,R=j,Z=0)\right) \end{matrix}$ (2)

An estimate of $\alpha$ can be obtained from (2) following these steps.

1. Estimate$\beta_{1}$,$\beta_{3}$,$\beta_{5}$and$\beta_{7}$ in model (1) as the empirical log odds of smoking in participants whose outcomes are known (that is, using the complete cases) using data from the groups *(X=0, Z=1)*, *(X=1, Z=1)*, *(X=0, Z=0)* and *(X=1, Z=1)* respectively.
2. Assume particular values of $\beta_{2}$,$\beta_{4}$,$\beta_{6}$and$\beta_{8}$, so that model (1) is identified. We use our estimates and assumed values of the eight$\beta$ parameters to estimate all $P(Y=1|X=i,R=j,Z=1)$ and $P(Y=1|X=i,R=j,Z=0)$ terms in the definition of $\alpha$.
3. Estimate the terms $P(X=i,R=j|Z=1)$ and $P(X=i,R=j|Z=0)$ as the empirical proportions of participants in the treatment and control groups that provide the required combinations of *X* and *R*.
4. All probabilities on the right hand side of the expression for $\alpha$ are now estimated. The estimate $\hat{\alpha}$ is obtained by replacing all these probabilities by their estimates and performing the calculation for $\alpha$ as shown.

If all even $\beta$ parameters are infinite then $\alpha$ can also be estimated by imputing missing outcome data as smoking or not smoking (table 3) and then evaluating the log odds ratio.
