# Supplementary material for: A general method for handling missing binary outcome data in randomized controlled trials
Source: Addiction. 2014 Nov 10;109(12):1986–93. doi: 10.1111/add.12721 (PMC4241048; doi:10.1111/add.12721)
Supplement: Appendix S3 — Further details of the spreadsheet. [file add0109-1986-SD3.zip › add12721-sup-0003-sm3.doc.docx]

**Further details of the spreadsheet:**

A purpose built spreadsheet has been created to implement the analyses. Recall that X=1 means that subjects are smoking at the previous time point, Y=1 means subjects are smoking at the end of the trial and R=1 means that Y is observed.

In the spreadsheet, the user provides (in the pale blue cells) the number of participants who belong to each (X, R) group for both treatment groups.

The user also provides (again in the in the pale blue cells) the number of subjects with Y = 1 in each (X, R = 1) group.

The user provides the assumed even numbered beta parameters in the red cells.

The yellow cells contain default values, which can be changed, to avoid numerical difficulties. If there are no participants who belong to a particular (X,R = 1) group then the corresponding odd numbered beta parameter is not estimable. The user may enter a minimum number of participants in groups which provide outcome data in cell B9 to avoid this problem in such instances. This replaces the size of such zero sized groups with this minimum number when analysing the data, so if cell B9 is nonzero it should be small. Similarly any zero counts of subjects who provide either Y = 0 or Y = 1 may be replaced by a `correction for zeroes' (cell B10) to avoid the problems associated with infinite log odds ratios. The value in B10 should be less than the entry in B9 in situations where these values need to be changed. Finally, infinite even numbered beta present problems for the spreadsheet so a maximum even numbered beta (100) is provided in cell B8. Since the values in B8 to B10 will change the values used in analysis from those that the user has provided if any of the above difficulties occur, the values used in analysis are shown in columns F-H, so that any necessary changes to the user’s inputs are transparent.

The inferences are shown in red font, at the bottom of columns A and B. Columns D and E show the smoking rates by subgroup and a short summary is shown at the bottom of columns F-H.
