# Supplementary material for: A general method for handling missing binary outcome data in randomized controlled trials
Source: Addiction. 2014 Nov 10;109(12):1986–93. doi: 10.1111/add.12721 (PMC4241048; doi:10.1111/add.12721)
Supplement: Appendix S4 — Example 2: the iQuit trial. [file add0109-1986-SD4.docx]

**Supplementary Materials: Example 2: the iQuit trial**

The iQuit trial is an internet based randomised controlled trial conducted among the general population of smokers seeking help from web-based resources. It assessed the effect on smoking cessation rates of self-help materials tailored to individual smoker characteristics compared with generic self-help materials. Participants filled in a questionnaire and received an online advice report to help them quit smoking. They were randomised to receive either the tailored version or the generic version. Six months later they received a telephone interview to find out whether they were still smoking, see if their smoking-related beliefs had changed at all, and find out what they thought of the advice they received. See Mason *et al*. for the analyses that we extend here via our sensitivity analysis.

There are 1758 participants in total, 1483 of whom were smoking at baseline (X=1), and 1036 participants have missing final smoking status outcome data. The pattern of missingness by treatment group is shown in table 7. This is a much larger trial with much lower response rates than our first example, but the proportion of missing data is similar in both treatment groups.

Table 7: The pattern of missingness for the iQuit data

Treatment Control Total

Smoking known 351 (40%) 371 (42%) 722 (41%)

Smoking unknown 526 (60%) 510 (58%) 1036 (59%)

Total 877 881 1758

In order to determine which values of even $\beta$ are plausible, noting that around 80% of participants in the treatment (smoking at baseline) are smoking at the end of the trial, the trialists were asked

“Suppose I told you that (of those smoking at baseline) in the treatment group of the iQuit trial, of those who did provide final outcome data, 80% were smoking at the end of the trial. What percentage of people in this group, who did not provide outcome data, do you think were smoking at the end of the trial?”

The trialists were asked to put a percentage weight on each of the percentages 0, 10, 20, …, 100% to reflect their beliefs about the missing data. They placed the greatest percentage weight (50) on 80% and percentage weights of 5, 35, and 10 on 70%, 90% and 100% respectively. They indicated that they interpreted the percentages of participants smoking as referring to ranges, rather than specific values, and that they did not consider “missing=smoking” very plausible. Interpreting $\beta_{3}$ as the log odds as explained in Table 1, equating this to logit(0.8/0.2) as in the phrasing of the question, equation (1) provides

$\text{logit P}\left(\text{Y}=\text{1 }|\text{ R=0},\text{ X=1},\text{ Z=1}\right)= \text{ logit(0.8/0.2) + }\beta_{4}\text{ }$ (3)

where X is the outcome at baseline. Hence using the elicited *P(Y=1|R=0, X=1, Z=1)*, plausible values of $\beta_{4}$ can be obtained from (3). We use three values of $\beta_{4}=-0.77,0,2.28$ in the sensitivity analysis because these correspond to *P(Y=1|R=0, X=1, Z=1)=0.65, 0.8, 0.975*, reflecting the range of values thought plausible. Three similar questions were then asked for the other combinations of *(X, Z)* to obtain plausible values for each of the other even numbered $\beta$ parameters.

These four questions do not provide any information about which combinations of even numbered $\beta$ parameters might be considered simultaneously plausible in the same model. In order to determine this, we asked the trialists which combinations of *P(Y=1|R=0, X=1, Z=1)* and *P(Y=1|R=0, X=1, Z=0)* they thought were plausible. This question was asked by providing a two by two grid showing the two sets of probabilities, with the accompanying percentage weights from the previous questions, and requiring that “Y” be put in cells where the two probabilities are simultaneously plausible. Nearly all “Y”s were placed along the main diagonal, leading to the conclusion that we should only consider models where $\beta_{4}=\beta_{8}$. However it was also thought possible that *P(Y=1|R=0, X=1, Z=1)* could be slightly less than *P(Y=1|R=0, X=1, Z=0)* in some instances, so some models where $\beta_{4}$is slightly less than $\beta_{8}$ could also be explored to reflect this belief.

An analogous question about the probabilities for those not smoking at baseline resulted in the conclusion that models where $\beta_{2}=\beta_{6}$ should be considered, but similarly some models where $\beta_{2}$ is slightly less than $\beta_{6}$ should also be explored. Further questions could also be asked to determine which combinations of other even $\beta$ parameters are simultaneously plausible. However we expect the possibility of different sensitivity parameters for the two treatment groups to have the most impact on the estimated treatment effect, so we have examined the most important issue. A variety, but not exhaustive, set of possibilities are explored in table 8.

Table 8: Results from the sensitivity analysis for the iQuit data. Standard errors are in parentheses.

$$\beta_{2}$$

$$\beta_{4}$$

$$\beta_{6}$$

$$\beta_{8}$$

$$\hat{\alpha}$$

-0.61 -0.77 -0.61 -0.77 -0.03 (0.18)

-0.61 0 -0.61 0 -0.00 (0.17)

-0.61 2.28 -0.61 2.28 0.06 (0.16)

0 -0.77 0 -0.77 -0.05 (0.18)

0 0 0 0 -0.02 (0.18)

0 2.28 0 2.28 0.05 (0.17)

2.54 -0.77 2.54 -0.77 -0.07 (0.18)

2.54 0 2.54 0 -0.05 (0.18)

2.54 2.28 2.54 2.28 0.01 (0.17)

Table 8 shows a distinct lack of evidence for a treatment effect for all values explored in the sensitivity analysis. This adds further weight to the conclusions from Mason *et al*. who report a lack of evidence of a treatment effect.

**Reference:**

Mason D, Gilbert H, Sutton S. Effectiveness of web-based tailored smoking cessation advice reports (iQuit): a randomised trial. Addiction 2012; 107 : 2183-2190.
